# The Nexus between Study Burnout Profiles and Social Support —The Differences between Domestic (Finnish) and International Master’s Degree Students

**DOI:** 10.3390/bs12030079

**Published:** 2022-03-15

**Authors:** Sara Rönkkönen, Markus T. Mattsson, Viivi Virtanen, Kirsi Pyhältö, Mikko Inkinen

**Affiliations:** 1 Faculty of Educational Sciences, University of Helsinki, 00014 Helsinki, Finland; markus.mattsson@helsinki.fi (M.T.M.); kirsi.pyhalto@helsinki.fi (K.P.); 2 The Centre for University Teaching and Learning (HYPE), Häme University of Applied Sciences (HAMK), 13101 Hämeenlinna, Finland; viivi.virtanen@hamk.fi; 3 Study and Career Counselling Psychologist Team, Aalto University, 00076 Aalto, Finland; mikko.inkinen@aalto.fi

**Keywords:** study burnout, social support, social well-being, higher education, learning and academic success, person-centric research

## Abstract

The present study investigated the variation in higher education students’ study burnout experiences and how they are related to academic success and social support needs. Similarities and differences between the international and domestic students were also explored. In this mixed-methods study, the data were collected through a self-reported questionnaire, and a total of 902 (response rate 42%) first year master’s students from the fields of arts, business and technology responded. Using Latent Profile Analysis (LPA), we detected three distinct study burnout risk profiles (No exhaustion or cynicism; Exhausted; Exhausted and cynical). The following distinct forms of social support needs were found using theory-based qualitative content analysis: informational, instrumental, emotional, and co-constructional support. We found out that the students with highest risk of burnout had the lowest grade point averages (GPAs). Further investigation showed that international students pass their courses despite the experiences of study burnout, even though the GPAs might deteriorate. When the domestic students experience study burnout symptoms, they both gain fewer study credits and earn lower GPAs. Finally, a relationship between the form of support needed and the burnout profile was identified.

## 1. Introduction

Higher education students’ study burnout has been identified as a serious study-related threat. Study burnout has shown to have severe consequences for both the individual and the society [1,2]. It has been shown to impair the student’s functioning, resulting in a decline in quality of health and learning [3] and developing depression later in life [1,4]. Study burnout is also related to life stress [5], decreased study achievement [6,7] and increased risk of dropping out [8]. The risk of developing burnout also seems to increase with years studied [2], and study-related exhaustion seems to grow during university studies [9]. In Finland, up to 11.5% (10.2% for men, 12.2% for women) of Finnish university students have been shown to suffer from an elevated risk of developing study burnout [10]. Recently, the lockdowns and social deprivation caused by the COVID-19 pandemic have further increased mental health concerns among higher education students globally [11,12,13].

There is body of evidence that social resources (such as getting support from one’s supervisor) and students’ ability to utilise them contribute to both reduced risk of suffering from study burnout and students’ study progress [14,15]. Social support has been shown to be negatively correlated with study burnout, especially with inefficacy experienced while studying [16]. In turn, a social strategy of being positively directed towards social relations and looking for social support and advice (high social optimism) has been found to predict lower levels of early career work burnout [2]. Moreover, higher education students’ ability to build and use social resources has been shown to be associated with reduced risk of developing study burnout [17,18].

Although individual consequences are associated with burnout while studying, we still do not understand individual variations in higher education students’ study burnout well enough [1]. Little is known about the inter-relation between the individual variations in study burnout and the support that higher education students would like to receive from the academy. Even less is known about similarities or differences between international and domestic students in this regard. To bridge the gap in the literature, in this study we have explored individual variations in study burnout and the social support needs reported by domestic (Finnish) and international master’s students, using a mixed method approach.

### 1.1. Study Burnout among HE Students and Comparing Domestic and International Students

*Study burnout* results from extensive and prolonged study-related stress [4]—see also seminal burnout-related work by Freudenberger [19],Maslach and Jackson [20]. It has three distinctive symptoms: *exhaustion*, referring to a lack of emotional energy in studies, feeling tense and tired in studies; *cynicism*, entailing losing interest and meaningfulness in one’s studies; and *inadequacy,* referring to sense of ineffectiveness and lack of accomplishment in one’s studying [1,21]. In full-blown study burnout, all the three symptoms might be experienced to a great extent [22,23].

Theoretically and methodologically, the burnout experience has been investigated from either variable or person-centred approaches. A variable-oriented approach focuses on the relations between variables and aims for generalizations [24,25]. A variable-oriented approach does not reveal individual variation nor bring out the heterogeneity of the burnout experience unlike a person-centred approach. The person-centred approach takes the individual as the unit of analysis and makes it possible to identify qualitative differences concerning experiences of burnout among people [26,27,28,29,30].

Even if the risk of developing study burnout is suggested to vary across individuals [27], studies focusing exclusively on individual differences in burnout experiences among higher education students are rare. Prior studies have typically clustered burnout with other constructs related to well-being whilst studying, such as study engagement [31] or coping behaviour [32]. The findings of the few person-centred prior studies on higher education students’ study burnout and engagement imply that consistent profiles, characterised by systematically high, moderate, or low levels of all burnout symptoms, and discrepant profiles entailing increased or high levels of one or two symptoms, can be identified [1]. However, the number of profiles detected has been shown to vary. For instance, Salmela-Aro and Read [1] identified four profiles of study engagement and burnout, namely “engaged”, “engaged-exhausted”, “inefficacious” and “burned-out”. They found that 7% of the students displayed the burned-out with low engagement and high cynicism profile. Portoghese, et al. [33], on the other hand, detected three profiles, including “burned-out”, “overextended” and “engaged” among Italian undergraduate and graduate students with 34.2% of the students presenting the “burned out” profile.

Results on the comparisons between international and domestic higher education students are somewhat inconsistent. Some studies implied that international students are more likely to experience study-related stress than the domestic ones [34], and that they may need different retention strategies [35]. However, in a comparative study on mental health, such differences between international and domestic students were not detected [36]. Moreover, the results from a few earlier studies comparing Finnish and international higher education students’ engagement and satisfaction towards studies have been contradictory. While Sakurai, et al. [37] showed that the engagement experienced by Asian and European students in the same educational context did not differ much, some other studies [38] reported that international students were more satisfied with their studying than Finnish students.

### 1.2. Social Support as a Buffer for Study Burnout

Social support refers to resources such as feedback from the university staff or peers, perceived to be available and to be used by higher education students (see seminal work by Cobb [39], House [40], House and Kahn [41]). Social support consists of both formal and informal relationships [42] within the study environment, including fellow students, lecturers, professors, and other members of the academic community, providing the primary sources of support for students (see Dupont, et al. [43]).

At least three distinct forms of social support for studying can be identified, including informational, emotional, and instrumental support [42,43,44]. Informational support is characterised as help in problem solving, such as guidance, feedback, affirmation, and advice. Emotional support refers to showing interest, listening, caring, and fostering the sense of belongingness, while instrumental support relates to resources like time, funding, materials, and equipment. In some prior studies, co-constructional support has also been identified as a fourth form of social support [44].

Social support has been shown to provide a resource for buffering burnout [45]. For example, receiving social support from several sources (having an extensive social support network) has been shown to reduce the risk of suffering from study burnout [16,44,46,47,48,49]. Additionally, there is evidence that receiving emotional and informational support for studying is related to satisfaction with supervision [50] and reduced risk of developing study burnout [51,52]. There is also tentative evidence that especially institutional support and support from the supervisors are associated with higher levels of motivation, which further predicts greater study engagement among higher education students [43].

## 2. Aims of the Study

Our aim with this study was to gain a better understanding of the individual variation in master’s students’ study burnout experience by identifying burnout risk profiles. Moreover, inter-relation between the profiles and academic success, as well as the need for social support, were examined. Similarities and differences between international and domestic students in these regards were also explored. The following research questions were addressed:

**RQ1.**  What study burnout risk profiles can be identified among 1st year master’s students?
**RQ2.**  Are the varied study burnout risk profiles related to study success (grades and study credits)? Do the international and the domestic students differ from each other in this regard?
**RQ3.**  What support needs did the students report?
**RQ4.**  Are there differences in reported needs for support between the students having different profiles of burnout risk? How do international cf. domestic students differ from each other in this regard?

## 3. Materials and Methods

### 3.1. Research Context

Research data were collected from first year master’s students in spring 2019 at a Finnish research-intensive university. In 2019, the total number of master’s students studying at the university was 5337, out of which 1360 were international students. The number of master’s degrees completed in 2019 was 1960. The target time for completing a master’s degree (120 ECTS credits, including the 30 ECTS credits thesis) is two years. In Finland, a student admitted to study only for a master’s degree has the right to complete the degree in four years and a student admitted to study both a bachelor’s and a master’s degree has seven years to complete both degrees. Since 2017, students from outside the EU/EEA area have been required to pay annual tuition fees to attend Finnish bachelor’s and master’s level programmes. Students liable for tuition fees may apply for scholarships that could waive the tuition fees fully or in part for the targeted time of the degree. The master’s students who took part in the study were either continuing from their bachelor’s, i.e., had been selected to complete both a bachelor’s and master’s degree through an entrance examination or, alternatively, had completed their bachelor’s or equivalent programme elsewhere and been selected into the master’s programme based on the institution’s entrance requirements.

### 3.2. Participants

A total of 902 (response rate 42%) master’s students from the fields of arts, business and technology responded to the questionnaire. The questionnaire was voluntary for the participants. When replying, the students were able to choose the language of the questionnaire between Finnish, Swedish and English. A total of 77% of the respondents were Finnish students (*n* = 699) and 23% international. A total of 61% of the domestic and 59% of the international students reported their gender as “male” (For gender distribution only binary “male” or “female” options were available in the database used). The participants represented well the whole master’s student population of the case university in terms of gender and the distribution of Finnish and international master’s students. The self-reported questionnaire data was combined with the study register data (grade point averages and number of credits (summer 2019). The participants were asked for their permission to connect their responses with data from the study register. The number of credits (ECTS) studied from the first academic year of the master’s studies varied from 0 to 99, and the mean number of credits studied was 46.5 (SD = 18.69). The grade point averages (GPAs) varied from 1.0 to 5.0 (on a scale from 1 to 5). The average GPA was 3.89 (SD = 0.69).

### 3.3. Study Design

Within this study, we used a convergent (concurrent) mixed-methods research design in which qualitative and quantitative data were collected simultaneously. Quantitative and qualitative analyses were conducted separately and merged afterwards [53,54].

#### 3.3.1. Measures

The mixed-methods data were collected in February 2019 by using an online study wellbeing inventory that is based on the HowULearn questionnaire validated in previous studies [55]. The participants were able to complete the questionnaire in Finnish, in Swedish or in English with either mobile, tablet or desktop user interfaces. The questionnaire link was sent three times to the participants belonging to the cohort group, and reminders to reply were sent via emails and text messages.

We used the (SBI-9) Student Burnout Inventory [3,56] to measure study-related burnout in the university context. It includes scales on *exhaustion* in higher education (four items), *cynicism* about the meaningfulness of studying (three items) and *sense of inadequacy* in studying in higher education (two items). See scales and items in Appendix A. The Student Burnout Inventory (SBI-9) has been validated in earlier studies [1,3,4]. All the items were rated on a 4-point Likert scale: *(1) I completely agree; (2) I agree to some extent; (3) I disagree to some extent; (4) I completely disagree*.

A qualitative measure for social support was utilized. The social support needed by the students was explored from the responses to an open-ended question, *“What changes or actions in teaching, supervision or services in your school, programme, or university would help you to improve your well-being?”*. A total of 532 students responded to the open-ended question (78% (*n* = 416) Finnish and 22% (*n* = 116) international students).

The background variables used were gender, nationality, accumulation of study credits in total (ECTS) and grade point average (GPA). Study success information (GPAs and credits) was gathered from the study register after the academic 2018–2019 year had ended, and the information of that year’s figures had been registered.

#### 3.3.2. Statistical Analysis

In this study, the burnout risk profiles were explored using Latent Profile Analysis (LPA which is a variant of Latent Class Analysis LCA). Students’ self-reported levels of *cynicism* and *exhaustion* were used as input sum variables in the present analysis, as the factor analysis conducted supported the use of these two sum variables. Parallel analysis was used to determine the number of factors to retain.

We used four model fit indexes: equal variance and shape with a diagonal distribution, oriented according to the coordinate axes (the EEI model); different variance and shape with a diagonal distribution, oriented according to the coordinate axes (the VVI model); equal variance and shape with an ellipsoidal distribution, equally oriented (the EEE model); and different variance and shape with an ellipsoidal distribution, variably oriented (the VVV model). For technical details of these models, see Scrucca, et al. [57].

Assessment of the question of the number of latent profiles to extract was based on the following statistical criteria: Log-likelihood, Bayesian Information Criterion (BIC), Akaike Information Criterion (AIC), Bozdogan’s consistent AIC (CAIC), Sample Size Adjuster BIC (SABIC), Integrated Complete Likelihood (ICL), Approximate Weight of Evidence (AWE), Classification Likelihood Criterion (CLC) and Kullback Information Criterion (KIC). In addition, the internal homogeneity of the classes was assessed using the entropy statistic (see Larose, et al. [58]) and the Bayesian Likelihood Ratio Test (BLRT) was performed to assess whether adding a further class to the model would provide a better fit to data than a model with one class fewer.

We used the multicriteria decision-making approach for deciding the number of latent profiles (the MCDM procedure; Peng, et al. [59]). More specifically, we applied the algorithm based on the Analytic Hierarchy Process [60], introduced by Akogul and Erisoglu [61], as a method for weighing the evidence produced by AIC, AWE, BIC, CLC and KIC. The final number of profiles was based on both the statistical indices and substantive criteria such as interpretability, parsimony, and theoretical understanding regarding the phenomenon of study burnout. In addition, graphical methods were used to illustrate the distributions of the indicator variables in the sample and the nature of the latent profiles.

The relationship between the latent burnout profiles and study success (grades and study credits) was examined by a series of analyses of variance (ANOVAs). First, differences between the profiles in study success were investigated by a one-way ANOVA, and second, the pattern of study success across profiles and the students’ country of origin were investigated using a two-way ANOVA. Effect sizes were quantified using the generalised eta squared statistic, which produces comparable results across various study designs. When calculating generalised eta squared, both independent variables (latent profile and students’ country of origin) were treated as observed rather than manipulated variables [62].

Finally, we investigated the relationship between the students’ burnout profile and the forms of support they reported needing. Because each student could report needing more than one form of support, statistical methods requiring the independence of observations (such as χ^2^ analysis) could not be used. The analysis question was thus conceptualised as a problem of Multiple Marginal Independence [63] and the statistical analyses performed using the MRCV package [64] in R.

#### 3.3.3. Qualitative Analysis

Students’ open-ended responses were analysed via theory-based qualitative content analysis [65,66]. Researcher Community and Supervisory Support Model [67]—see also Väisänen, Pietarinen, Pyhältö, Toom and Soini [15], Cornér, et al. [68]—was applied in analyses of master students’ support needs in terms of the forms and sources of support for studying. The qualitative data set consisted of approximately 30,000 words.

The data were coded into two categories based on the *form* of support and the *sources* of support. The different forms of support included (a) *emotional* support, comprised of descriptions of lack of or a need for encouragement, trust, showing interest and a sense of belonging; (b) *informational* support, including reports of a lack of or a need for informative advice, (pedagogical) expertise, guidance, and feedback; (c) *instrumental* support, entailing expressions of a lack of or need for support in the form of time, materials funding and networks; and (d) *co-constructional* support, including descriptions of co-creation of new knowledge. Five categories of support *source* emerged from the data: (1) *Teacher,* meaning any member(s) of the academic staff involved in teaching, including hourly paid teachers, lecturers, professors, academic advisors, etc.; (2) *Peers,* meaning fellow students; (3) *Support services* such as study psychologists, administrative services, and other study counselling; (4) *Organisational level,* including the structures of the university, department, study programme or equivalent; (5) *Academic community* at large. The first and third authors oversaw the coding and justifications, and decisions of the classification principles were discussed, verified, and validated within the research group. Finally, the qualitative data were converted into quantitative form by coding the qualitative data and counting the frequencies.

## 4. Results

### 4.1. Determining the Latent Study Burnout Risk Profiles

Latent profile models comprised of 2–9 profiles and different within-profile covariance structures were explored based on the statistical and substantive criteria described in Section 3.3.2.

As Table 1 shows, several of the statistical criteria favoured a model with three or five profiles based on the within-profile covariance structure VVV (variable volume, shape, and orientation). The criteria AIC, CLC and KIC favoured the VVV5 model, whereas BIC, CAIC and SABIC favoured the VVV3 model. The Analytic Hierarchy Process, which was described in Section 3.3.2, favoured the VVV3 model. Looking at the results of the Bayesian Likelihood Ratio Test (BLRT) for the models that are based on the within-profile covariance structure VVV, the model with two profiles fit better than the model with only one large profile, the model with three profiles fit better than the model with two profiles, and the model with four profiles did not fit better than the model with three profiles. Thus, when working within the within-profile covariance structure VVV, the BLRT favoured the model with three profiles. Judged by the entropy statistic, the profiles in the three (VVV3) and five (VVV5) profile models were roughly equally homogenous, even though the model with three profiles had a slightly higher entropy (indicating more homogenous classes). Among these alternatives, the statistical evidence thus favoured the model with three profiles, which was also parsimonious and interpretable. Accordingly, aligned with the theoretical construct of the burnout syndrome [1,21], the three burnout profiles detected differed from each other in regard to both the primary burnout symptom(s) characterizing the profiles and the intensity of the symptom(s) reported.

### 4.2. The Study Burnout Risk Profiles

**Figure 1:**
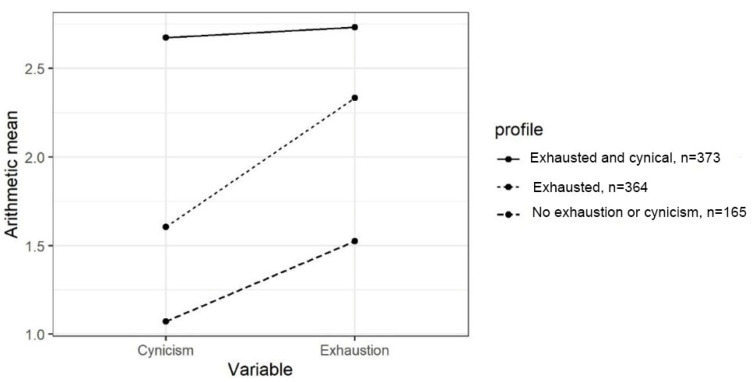
The group means for the indicator variables for study-related burnout profiles.

The group means for the indicator variables for the latent profiles are shown in Figure 1. The profiles were named *Exhausted and cynical*, *Exhausted,* and *No exhaustion or cynicism*. Students belonging to the *Exhausted and cynical* profile showed both high levels of exhaustion and high levels of cynicism; this profile comprised 41.5% of the students, *n* = 373. Compared to other profiles’ students, the *Exhausted and cynical* profile showed the highest levels of both burnout symptoms. Students belonging to the *Exhausted* profile displayed quite low levels of cynicism but elevated levels of exhaustion; this profile comprised 40.5% of the students, *n* = 364. Students in profile *No exhaustion or cynicism* comprised 18% of the sample, *n* = 165. This profile was characterised by low levels of exhaustion experienced and very low levels of cynicism, indicating no risk for burnout compared to other two profiles.

There were no statistically significant differences between the profiles in which the Finnish and international students fell. The differences in the proportions were also small when assessed using *Cohen’s w* as a metric for effect size (*p* = 0.193, *w* = 0.086).

### 4.3. Study Success and Study Burnout Risk Profiles: Comparing Domestic and International Students

The relationship between study burnout risk profile membership and study success (credits per year and GPAs) was examined using a series of ANOVAs. First, course credits and GPAs were compared across profiles in a one-way ANOVA.

The students earned roughly equal amounts of study credits across the three profiles, but their GPAs differed in a statistically significant manner as shown in Table 2.

**Figure 2:**
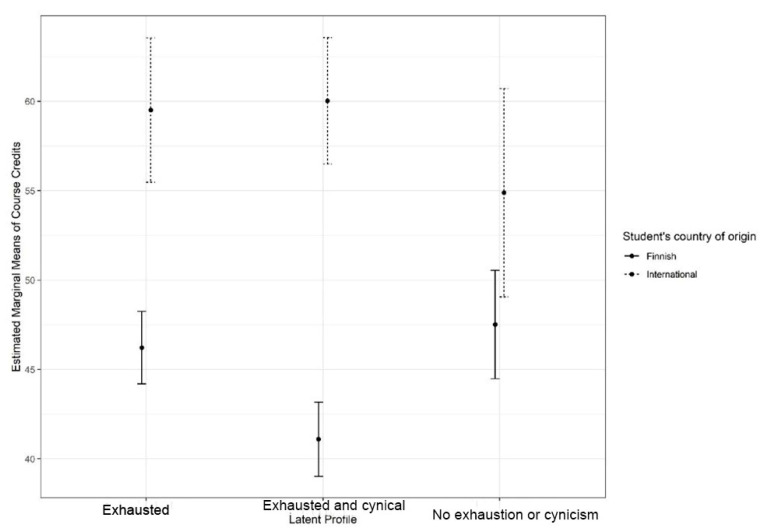
Credits. Means of course credits across the latent profiles for domestic and international students with 95% confidence intervals.

We then examined the interaction of the international status of the students and study success across the profiles. There was a significant interaction between the students’ international status and profiles on study credits, F (2.896) = 4.62, *p* = 0.01, generalised eta squared = 0.009. Simple main effect analysis showed that the numbers of study credits earned differed only within the group of domestic students (*p* < 0.001 and *p* = 0.318, for Finnish and international students, respectively). Accordingly, Finnish students belonging to the *Exhausted and cynical* profile earned significantly fewer study credits than Finnish students belonging to the other two profiles; similar differences were not detected within the group of international students. The results are shown in Figure 2.

**Figure 3:**
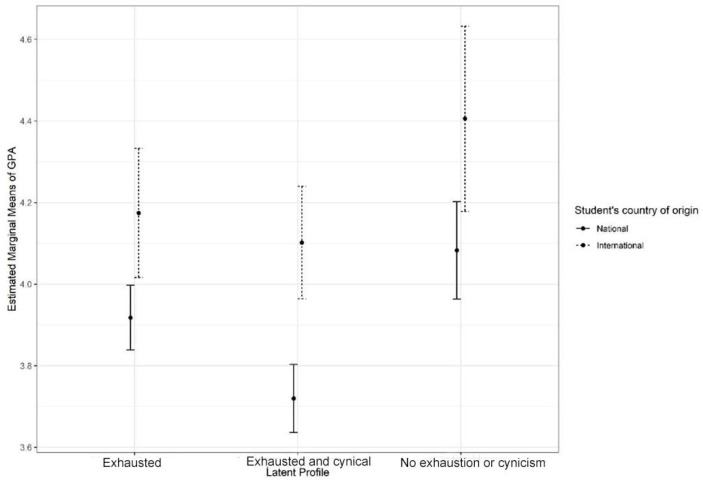
Means of GPAs across the latent profiles for domestic and international students with 95% confidence intervals.

When looking at the GPAs in terms of the students’ international status and profiles, no interaction effect was found, F (2.871) = 0.53, *p* = 0.589, generalised eta squared = 0.001. However, there was a main effect of international status, F (1.871) = 28.97, *p* < 0.001, generalised eta squared = 0.031. The results are shown in Figure 3.

It is of interest to compare the pattern of results concerning study credits and GPAs: when it comes to the former, the interaction between the burnout profile and the student’s international status was significant, whereas when it comes to the latter, it was not. The main effect of burnout profile was still significant, reflecting the fact that students belonging to burnout profile *Exhausted and cynical* had the lowest GPAs, students belonging to profile *No exhaustion or cynicism* the highest GPAs, and students belonging to profile *Exhausted* something were in between the other two profiles.

### 4.4. The Social Support Needed: Forms and Sources

The students described *informational, instrumental, emotional,* and *co-constructional support* needs. However, the participants rarely reported lack of support related to the latter form of support. Table 3 shows the five support *sources* identified from the data and their relation to the support *forms* recognised.

In the following text, we present representative quotes from each category of the support forms identified (I = international, D = Domestic, all quotes translated into English by the fist author):

*Informational support* (f = 281; f(%) = 39.7%) such as need for informative advice, (pedagogical) expertise, guidance, feedback, affirmation, and help in problem-solving was most frequently reported by the participants. For instance, they described the need for well explained information about the services and courses as well as high quality instruction as a means for improving their study well-being. Most often, the students reported a need to receive feedback from their professors:

*“I feel like X offers a very wide range of services, because I keep hearing about new things from students around me, but sometimes I’m just not quite sure where to find them or what they even are, so being informed of them more would be great.”* (I_3134)

*“Finding interesting courses is a challenge. The database is full of similar names to which the same jargon has been copied. Only information from older students through a bush radio tells you what’s going on.” * (D_168)

*“Teachers seem too busy and you don’t get proper feedback from them.”* (D_174)

*“I think pedagogy could be improved. It would be good to teach things by repeating the basics. Sometimes it feels like the courses rely too much on the teachings of the previous course. This could be improved, for example, by providing a review of the necessary basic issues as additional material. In lectures, new things seem to come up exponentially, so it would be good to repeat previous lectures of the same course (even in a few sentences). And connects lecture issues with (practical) examples.”* (D_121)

Students also highlighted *instrumental support* (f = 267; f(%) = 37.7%) entailing time, materials funding, networks, etc. to be important for their study well-being. They typically described a need for organisational level tangible forms of help such as suitable facilities, or sufficient time allocation that would enable students to manage their studies properly:

*“[…] I’m limited in the choice of courses as I don’t have much time as other students who are free to study up to 4 years. Besides, if I want to devote a whole year for my thesis writing like other EU students, I have to finish all of my courses from the first year, which is impossible. […]”* (I_1000)

*“To plan better the amount of work in each course considering all the courses that we have to take each period in a mandatory way.”* (I_ 3367)

*“I really appreciate having video recordings of lectures in order to have more options on dealing with the pressure of continuous deadlines.”* (I_ 3577)

*“An idea: The definition of a credit could be changed, as 15 ECTS/period means a rather inhumane working week. Another option is to stretch the periods. On the one hand, it is good that students are required to work hard and learn, but on the other hand, it does not make sense for so many to take much more than 5 years to complete their studies.”* (D_ 3639)

*Emotional support* consisting of lack of encouragement, trust, showing interest and a sense of belonging, (f = 151; f(%) = 21.3%) was less often described. The lack of support in this aspect was most often addressed to teachers. Students, for example, emphasised the importance of being heard and their work being acknowledged by their professors:

*“We had issues with professor before that also who wasn’t capable enough to listen and have the time and willingness to answer as well. We filed complaint even but nothing changed […]”* (I_1324)

*“In general, some mandatory one-on-one well-being consulting can be useful. In my case, I would probably not go to a consultation meeting voluntarily.”* (I_3224)

Students rarely expressed a need identified as co-constructional support (f = 9; f(%) = 1.23%) such as engaging in collaborative thinking and shared knowledge construction when doing groupwork. However, when they did, it was perceived as being central to learning and support for orchestrating the co-constructional activities:

*“In group work, competence remains one-sided and often insufficient background support from the course organizers leads to a loss of motivation for the whole group. […]. Learning also often plays a side role in group work, as usually the areas of responsibility are divided according to previous competence, so it is not possible to develop competence in other subject areas.”* (D_3438)

### 4.5. Forms of Social Support Needed and Study-Related Burnout Risk Profiles

The relationship between the three forms of social support needed (Table 3) and the three study burnout profiles (Figure 1) were analysed as a problem of Multiple Marginal Independence (see Section 3.3.2). The procedure is based on representing the relationships as several cross-tabulations (see Table 4 below). As the frequencies of co-constructional support were low compared to other sources of support, this form of support was excluded from the analysis.

**Figure 4:**
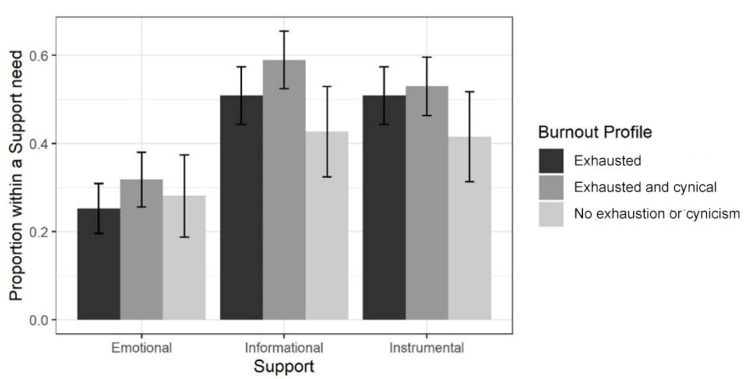
Support needs within burnout profiles. The lines indicate 95% confidence intervals that were calculated using the asymptotic method (normal approximation).

Results of the MMI analysis on support needs across the burnout profiles indicated that there was a relationship between the form of support required by the students and their burnout profile (bootstrapped *p*-value: 0.0434; second-order adjusted *p*-value: 0.0460). The results were further investigated by inspecting the individual χ^2^ test values in the subtables concerning each form of support; there, the test statistics receive the values 2.36, 7.31 and 3.37, and the Cramer’s V values of 0.067, 0.117, 0.080, respectively. This indicates that the relationship between requiring support and the burnout profile was strongest for a need of *informational* support. Based on Figure 4, it appears this is because especially students belonging to burnout profile 2 *Exhausted and cynical* required informational support, whereas students belonging to burnout profile 3 *No exhaustion or cynicism* did not do so. The need for all forms of support was highest among those students who belonged to the 2 *Exhausted and cynical* profile.

### 4.6. Forms of Social Support Needed and Study-Related Burnout Risk Profiles: Comparing Finnish and International Students

We proceeded by examining the relationship between support needs within each burnout profile, comparing the support needs of Finnish and international students. Results of the MMI analysis on the support needs of Finnish cf. international students across the burnout risk profiles indicated significant relationships only within profile, *Exhausted*: bootstrapped *p*-value 0.001; second-order adjusted *p*-value 0.0006.

**Figure 5:**
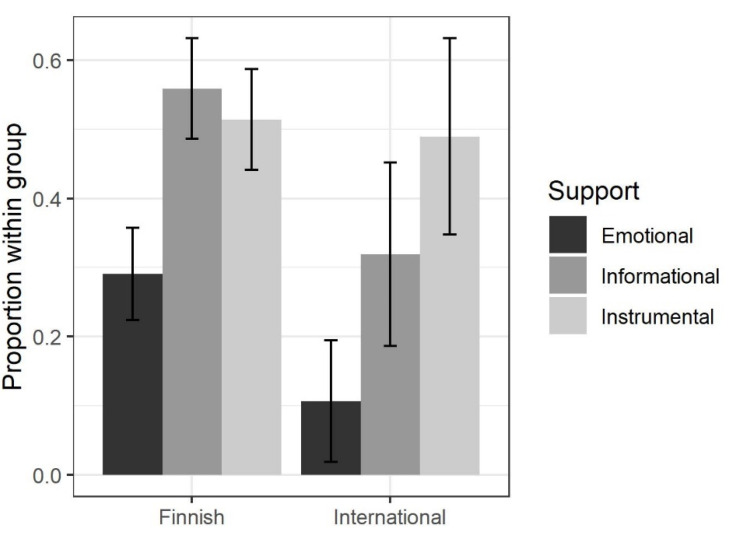
Support needs of Finnish and international students belonging to profile *Exhausted*.

Within profile *Exhausted*, the individual χ^2^ test values in the subtables of Finnish and international students were 6.69, 8.54 and 0.09, and the Cramer’s V values 0.159, 0.184 and 0.009, respectively. This indicates that Finnish and international students belonging to that profile differed in their needs for emotional and informational support (Finnish students required more emotional and informational support than international students did), while their instrumental support needs were roughly equal. These results are illustrated below in Figure 5.

## 5. Discussion

This study aimed to gain a better understanding on variation in higher education students’ study burnout experiences and how different experiences of burnout are related to academic success and social support needs. Similarities and differences between the international and domestic first year master’s students were also explored.

We detected three distinct study burnout risk profiles, including *Exhausted and cynical* profile (41.5%) with high levels of exhaustion and high levels of cynicism, *Exhausted* profile (40.5%), with quite low levels of cynicism, but elevated levels of exhaustion, and *No exhaustion or cynicism* profile (18%) with low levels of exhaustion and very low levels of cynicism. The findings are partly aligned with prior person-centred studies on higher education students’ study burnout experience in terms of the number and nature of profiles detected (see Salmela-Aro and Read [1], Lee, et al. [69],Salmela-Aro and Upadyaya [70]).

Interestingly, students earned roughly equal numbers of study credits across the three burn out risk profiles, but their grade point averages (GPAs) differed in a statistically significant manner: the students with highest risk of burnout had the lowest GPAs, while the students with the lowest risk had the highest GPAs. Accordingly, burnout risk seems to be associated with academic achievement. Further investigation showed that there was a significant interaction between the students’ international status and burn out risk profiles on study credits. However, the amounts of study credits earned differed only within the group of domestic students: Finnish students belonging to the *Exhausted and cynical* earned significantly fewer study credits than Finnish students belonging to the other two profiles; similar differences did not emerge within the group of international students. The results suggest that the international students succeed in passing their courses despite their experiences of study burnout, even though their GPAs might deteriorate; on the other hand, when the Finnish students show more severe burnout symptoms, they both pass fewer courses and earn lower GPAs. A reason for this might be that international students’ motivation (see e.g., Chue and Nie [71]) and, e.g., financial pressure to complete the master’s degree in two years is stronger than that of the native students and may lead to degree completion within the target time at the expense of the quality of learning.

The students reported four distinct social support needs, including *informational* support, *instrumental* support, *emotional* support, and *co-constructional* support that would enhance their study well-being. Based on our findings both the domestic and international students pointed out the need for feedback from their teachers as well as good instructions and guidance for studying. One big issue that emerged from the data was the need for time and resources such as recorded lectures to help in time-management to proceed in one’s studies. Accordingly, particularly informational, and instrumental support were emphasised by the students. The finding is in line with prior studies suggesting that higher education students expect to receive particularly informational and instrumental support from the teaching staff [15,72]. At the same time, students seldom expressed a need for emotional support or co-constructional support. A reason for this might be that their relationships with the teaching staff is not close enough to express such needs. This concerns also co-construction that usually requires long term close collaboration to occur. The most common sources from which the support was expected were teachers, the organisational level, and support services. Peers and academic community were less often reported as sources of social support, which might have been partly due to the formulation of the open question used for data collection. However, it is of interest that at the same time as the scholarly community and peers are unrecognised as a source of support, there is high demand for informational support targeted towards teachers. The finding suggests that while facing stressors in studying, the students expect support primarily from the formal institutional support providers. This implies that developing institutional support resources, including teachers’ competences to consider study wellbeing as part of the high-quality teaching, might be a good investment.

Moreover, a relationship between the form of support needed and the burnout profile was identified. The need for all forms of support was highest among those students who belonged to the *Exhausted and cynical* profile. The relationship between requiring support and the burnout profile was strongest for a need of *informational* support. This is because students, especially those belonging to *Exhausted and cynical* profile, required informational support, whereas students belonging to burnout *No exhaustion or cynicism* profile did not do so. Accordingly, our findings imply that social support does provide an important resource for enhancing study wellbeing among higher education students. Particularly, informational support seems to play a key role not only in cultivating study progress but also potentially on burnout prevention. Variation across the profiles in reported support needs, however, also implies that more individualized support practices should be designed depending on the students’ needs.

When comparing the support needs of Finnish and international students within each burnout profile, the results showed significant difference only within one profile. Finnish and international students belonging to the *Exhausted* profile differed significantly in their emotional and informational support needs, while their needs for instrumental support were roughly equal. With this profile, in which student’s cynicism is still quite low, the Finnish students required more emotional and informational support than international students did. It is worth questioning where the difference comes from. Are the moderately exhausted international students aware enough of the opportunities to ask for emotional and informational support in the Finnish academic culture? There is previous evidence that international students have higher levels of intrinsic motivation than domestic students [71]. Could international students’ higher motivation and other potential individual differences in self-regulation, study strategies, resilience, and strong agency act as buffers against study burnout even without the social support? The finding also implies that different student groups might benefit from varied forms of support in burnout prevention. Moreover, the finding indicates that the most functional support form may vary depending on the primary symptom of burnout displayed by the student.

The cross-sectional setting of this study means that we are not able to comment on the sequential hypotheses concerning the burnout experience. However, the profiles found might resonate with the sequential conceptual model [23] about the development of burnout in which exhaustion, caused by overload and too high demands, is assumed to develop first, precipitating detachment and negative reactions towards others and the task at hand (cynicism). Additionally, the results support recent research according to which the experience of cynicism may be more of a core part of burnout when compared with exhaustion [23]. Naturally, this viewpoint would require a separate study with a different study design.

### 5.1. Implications for Developing Higher Education in Finland

This person-centred study provides evidence that higher education students’ experiences of burnout vary. Understanding the variation and how different experiences of burnout are related to academic success and social support needs should be considered when planning teaching and support services and creating supportive and inclusive academic culture. The results in differences between international and domestic higher education students should be considered when designing education. The curricula should be designed in a way that all degree students can graduate in a targeted amount of time without burning out. Maybe the educators are not aware of the potential difficulties that international students experience (see also Hendrickson, et al. [73]), and they may not have the skills and tools needed to support and facilitate international students’ learning. Additionally, it is worth questioning whether the amount of study credits per year should be considered as an indicator of academic success among international students at all. It seems that the international students succeed in completing the required annual amount of study credits, even though their GPAs might deteriorate and despite their experiences of study burnout. Intriguingly, when the students were asked to tell what would increase their wellbeing, the support needs that they reported were directly related to studying. Would this indicate that enhancing wellbeing requires developing the basic processes of teaching and learning that either increase or decrease wellbeing?

In the funding model of the Ministry of Education and Culture in Finland for 2021–2024, 56% of the funding of universities of applied sciences (up from 40%) and 30% of the funding of universities (up from 19%) depends on the number of degrees they produce. In practice, this means that in the future, faster graduation and more efficient use of university places will be emphasised even more than thus far. Investing in wellbeing is investing in successful and efficient studying, but on the other hand, well-functioning educational practices also improve wellbeing. Additionally, in the Finnish Ministry of Education and Culture’s *Vision for higher education and research in 2030*, one of the targets is to increase the number of students pursuing a degree in higher education (up to 50% of the age group by 2030). All this will require more focus on higher university pedagogics, guidance, inclusion, and support practices.

### 5.2. Limitations of the Study and Future Research

The following limitations should be considered in any possible intention to generalise the results of this study. First, the study was cross-sectional, and thus there is also a need for a longitudinal study in the higher education context. Second, this study was carried out only in one country and in a single higher education institution, covering only specific fields of study. Generalising the results to higher education contexts in other countries would need to be critically considered, and further research is needed. Third, even if the response rate was moderate, it should be emphasised that the results cannot be generalised as such to the entire population from which the sample was drawn. The results are applicable specifically to those who responded. Fourth, the data were collected through a self-reported questionnaire survey that is conducted only once during the academic year. It is possible that the results could have been different if measurement had occurred at different time of the year, and self-reporting is subject to several biases. For future research, it would be valuable to combine self-report data with other information, such as an individual’s behaviour or physiological data. It is also worth emphasising that even if the *Social support model* used as a conceptual framework for framing the data analysis fitted well for the data, the model has been developed in the context of PhD students and postdoctoral students.

## Figures and Tables

**Table 1:** Model fit for the latent profile models with different numbers of profiles and different within-profile covariance structures.

Model	Classes	LogLik	AIC	AWE	BIC	CAIC	CLC	KIC	SABIC	ICL	Entropy	BLRT	BLRT, p
EEI	2	−1986.19	3986.38	4087.19	4020.01	4027.01	3973.83	3996.38	3997.78	−4191.39	0.73	335.68	0.01
EEI	3	−1931.96	3883.92	4028.49	3931.97	3941.97	3865.45	3896.92	3900.21	−4163.54	0.76	108.46	0.01
EEI	4	−1916.49	3858.98	4047.54	3921.44	3934.44	3834.34	3874.98	3880.15	−4330.46	0.68	30.94	0.01
EEI	5	−1878.84	3789.67	4021.84	3866.55	3882.55	3759.25	3808.67	3815.73	−4208.50	0.79	75.31	0.01
EEI	6	−1900.06	3838.12	4114.3	3929.41	3948.41	3801.51	3860.12	3869.07	−4421.09	0.7	−42.45	0.98
EEI	7	−1897.98	3839.95	4159.99	3945.66	3967.66	3797.33	3864.95	3875.79	−4511.03	0.69	4.16	0.21
EEI	8	−1860.60	3771.2	4134.9	3891.32	3916.32	3722.73	3799.2	3811.92	−4310.98	0.76	74.76	0.01
EEI	9	−1859.48	3774.96	4182.56	3909.49	3937.49	3720.42	3805.96	3820.57	−4437.79	0.73	2.24	0.26
VVI	2	−1935.29	3888.58	4018.52	3931.83	3940.83	3872.13	3900.58	3903.24	−4070.45	0.77	437.47	0.01
VVI	3	−1866.56	3761.12	3964.2	3828.38	3842.38	3734.57	3778.12	3783.92	−4093.05	0.73	137.46	0.01
VVI	4	NC	NC	NC	NC	NC	NC	NC	NC	NC	NC	NC	NC
VVI	5	NC	NC	NC	NC	NC	NC	NC	NC	NC	NC	NC	NC
VVI	6	NC	NC	NC	NC	NC	NC	NC	NC	NC	NC	NC	NC
VVI	7	NC	NC	NC	NC	NC	NC	NC	NC	NC	NC	NC	NC
VVI	8	NC	NC	NC	NC	NC	NC	NC	NC	NC	NC	NC	NC
VVI	9	NC	NC	NC	NC	NC	NC	NC	NC	NC	NC	NC	NC
EEE	2	−1942.51	3901.03	4016.45	3939.47	3947.47	3886.48	3912.03	3914.06	−4110.28	0.72	111.87	0.01
EEE	3	−1942.68	3907.35	4067.16	3960.2	3971.2	3886.25	3921.35	3925.27	−4640.47	0.45	−0.32	0.73
EEE	4	−1941.50	3910.99	4114.73	3978.26	3992.26	3883.79	3927.99	3933.79	−4975.53	0.4	2.36	0.1
EEE	5	−1895.72	3825.44	4072.37	3907.11	3924.11	3792.86	3845.44	3853.12	−4322.48	0.71	91.56	0.01
EEE	6	−1895.02	3830.04	4120.87	3926.13	3946.13	3791.39	3853.04	3862.61	−4457.48	0.68	1.4	0.37
EEE	7	−1893.35	3832.69	4167.36	3943.2	3966.2	3788.04	3858.69	3870.16	−4546.15	0.67	3.34	0.35
EEE	8	−1859.46	3770.91	4149.24	3895.83	3921.83	3720.43	3799.91	3813.26	−4328.19	0.76	67.78	0.01
EEE	9	−1858.26	3774.52	4196.73	3913.86	3942.86	3717.98	3806.52	3821.76	−4440.08	0.73	2.39	0.23
VVV	2	−1902.53	3827.05	3986.43	3879.9	3890.9	3806.38	3841.05	3844.97	−4104.12	0.66	191.84	0.01
VVV	3	−1853.80	3741.61	3988.65	3823.28	3840.28	3708.92	3761.61	3769.29	−4175.43	0.66	97.45	0.01
VVV	4	−1847.55	3741.1	4075.81	3851.61	3874.61	3696.41	3767.1	3778.56	−4325.32	0.65	12.5	0.14
VVV	5	−1834.97	3727.93	4150.31	3867.27	3896.27	3671.23	3759.93	3775.17	−4382.59	0.65	25.17	0.01
VVV	6	NC	NC	NC	NC	NC	NC	NC	NC	NC	NC	NC	NC
VVV	7	NC	NC	NC	NC	NC	NC	NC	NC	NC	NC	NC	NC
VVV	8	NC	NC	NC	NC	NC	NC	NC	NC	NC	NC	NC	NC
VVV	9	NC	NC	NC	NC	NC	NC	NC	NC	NC	NC	NC	NC

NC = The analysis did not converge for the model in question.

**Table 2:** Study credits and GPAs (grade point averages) across profiles.

	Exhausted *n* = 364	Exhausted and Cynical *n* = 373	No Exhaustion or Cynicism *n* = 165	F-Value	*p*-Value	Effect Size (Generalized Eta Squared)
Study credits	48.9 (18.1)	45.9 (19.8)	49.1 (17.5)	2.87(df 2.899)	0.057	0.006
GPA	3.97 (0.69)	3.82 (0.76)	4.15 (0.64)	12.962 (df 2.874)	<0.001	0.029

**Table 3:** The percentages of different sources of support in each support form.

	From Teacher f(%)	From Organisation f(%)	From Support Services f(%)	From the Academic Community f(%)	From Peers f(%)	From Other Sources f(%)
Informational (f(%) = 39.7%)	55.5%	27.3%	16.3%	0.6%	0.3%	-
Instrumental (f(%) = 37.7%)	25.8%	63%	1%	-	0.7%	9.5%
Emotional (f(%) = 21.3%)	42.2%	33.7%	10.2%	9.6%	3.6%	-
Co-constructional (f(%) = 1.23%)	50%	-	-	10%	40%	-

**Table 4:** Forms of support reported by students (A = domestic students, B = international students) belonging to each of the burnout profiles 1 = Exhausted; 2 = Exhausted and cynical 3 = No exhaustion or cynicism.

	Informational		Instrumental		Emotional	
	A	B	A	B	A	B
**1**	111	115	111	115	169	57
**2**	89	128	102	115	148	69
**3**	51	38	52	37	64	25

## Data Availability

Not applicable.

## Appendix A

**Table A1:** SBI-9 scales and items.

Exhaustion	Cynicism	Inadequacy
1. I feel overwhelmed by the work related to my studies 2. I often sleep badly because of matters related to my studies 3. During my free time I worry over matters related to my studies 4. The pressure of my studies causes problems in my close relationships with others	1. I feel a lack of study motivation and often think of giving up 2. I feel that I am losing interest in my studies 3. I am continually wondering whether my studies have any meaning	1. I often have feelings of inadequacy in my studies 2. I used to expect I would achieve much more in my studies than I expect now
